# Roxarsone induces angiogenesis via PI3K/Akt signaling

**DOI:** 10.1186/s13578-016-0119-1

**Published:** 2016-09-28

**Authors:** Yujing Wang, Donglai Yin, Chao Xu, Kai Wang, Lingmin Zheng, Yumei Zhang

**Affiliations:** 1 Department of Veterinary Pharmacology and Toxicology, College of Veterinary Medicine, Yangzhou University, 12# Wenhui East Road, Yangzhou, 225009 Jiangsu China; 2 Department of Veterinary Internal Medicine, College of Veterinary Medicine, Yangzhou University, Yangzhou, Jiangsu China; 3 Jiangsu Co-innovation Center for Prevention and Control of Important Animal Infectious Diseases and Zoonoses, Yangzhou, China

**Keywords:** Angiogenesis, Roxarsone, PI3K/Akt signaling, Vascular endothelial cell, B16–F10 xenograft model

## Abstract

**Background:**

3-Nitro-4-hydroxy phenyl arsenic acid, roxarsone, is widely used as an organic arsenic feed additive for livestock and poultry, which may increase the level of arsenic in the environment and the risk of exposure to arsenic in human. Little information is focused on the angiogenesis roxarsone-induced and its mechanism at present. This paper aims to study the role of PI3K/Akt signaling in roxarsone-induced angiogenesis in rat vascular endothelial cells and a mouse B16–F10 melanoma xenograft model.

**Results:**

The results showed that treatment with 0.1–10.0 µmol/L roxarsone resulted in an increase in the OD rate in the MTT assay, the number of BrdU-positive cells in the proliferation assay, the migration distance in the scratch test and the number of meshes in tube formation assay. Further, treatment with 1.0 µmol/L roxarsone was associated with significantly higher phosphorylation of PI3K/Akt and expression of VEGF than the control treatment. The PI3K inhibitor was found to significantly combat the effects of 1.0 µmol/L roxarsone. Furthermore, roxarsone treatment was observed to increase the weight and volume of B16–F10 xenografts and VEGF expression and PI3K/Akt phosphorylation in a dose-dependent manner, with the 25 mg/kg dose having significant effects.

**Conclusions:**

These results demonstrate that roxarsone has the ability to promote growth and tube formation in vascular endothelial cells and the growth of mouse B16–F10 xenografts. Further, the findings also indicate that PI3K/Akt signaling plays a regulatory role in roxarsone-induced angiogenesis in vivo and in vitro.

## Background

3-Nitro-4-hydroxy phenyl arsenic acid, also known as roxarsone, is widely used as an organic arsenic feed additive for livestock and poultry. Only a small amount of roxarsone that in ingested in animals is absorbed by the body, which means that the vast majority of roxarsone is excreted via the feces [1, 2]. Thus, the use of animal manure as an organic fertilizer is associated with an increase in the level of arsenic in the environment and a subsequent increase in the risk of arsenic exposure in humans [3, 4]. There is a high correlation between the level of arsenic in environment and the risk of human cancers including skin, bladder, lung, and liver cancers [5].

Arsenic has been identified as a type II carcinogen by the International Agency for Research on Cancer [6]. On the other hand, certain arsenic compounds have been approved by the Food and Drug Administration for the treatment of some blood cancers such as acute promyelocytic leukemia and their efficacy has been evaluated in clinical trials of the treatment of multiple myelomas and a variety of solid tumors [7]. Arsenic is known to have a biphasic impact on angiogenesis: that is, it promotes angiogenesis at low levels and inhibits angiogenesis at high levels. This may explain the controversial biological effects of arsenic on cancers. For example, Nicole et al. [8] suggested that the levels used clinically to treat leukemia may not be suitable for treating solid tumors, since the therapeutic levels for leukemia may enhance solid tumor growth instead of limiting it.

Roxarsone is widely used as a growth promoter to improve weight gain, feed efficiency and pigmentation in chicken and pigs. The mechanism by which roxarsone increases productivity is not yet clear, but increased blood supply (venous stasis) is recognized as a factor that promotes coloration [9]. Basu et al. [10] reported that low concentrations of roxarsone had a higher angiogenic index than As^III^ in human aortic endothelial cells and lung microvascular endothelial cells. Their results also indicated that roxarsone and As^III^ promote the angiogenic phenotype in human endothelial cells through their distinct effects on signaling mechanisms. Our previous studies have also demonstrated the potential of roxarsone to promote angiogenesis in vitro in rat endothelial cells and ex vivo in rat aortic rings [11, 12]. Angiogenesis plays a pivotal role in the initiation of carcinogenesis and tumor progression, vascular diseases and various ischemic and inflammatory diseases [13–16]. The pro-angiogenic effect of roxarsone may explain why arsenic exposure is closely associated with the development of many cancers [17].

Phosphatidylinositol-3-kinase (PI3K) is located at the cell membrane; when it is activated by a number of receptor tyrosine kinases, it initiates the signaling cascade via phosphorylation of phosphatidylinositol-4,5-diphosphate (PIP2), which leads to accumulation of phosphatidylinositol-3,4,5-triphosphate (PIP3). This secondary lipid messenger recruits Akt to the cell membrane, where Akt is phosphorylated and regulates cellular processes by activating a number of substrates, such as the mammalian target of rapamycin (mTOR), BclxL/Bcl-2-associated death promoter (BAD), murine double minute (MDM2) and nuclear factor κB (NF-κB) [18, 19]. PI3K/Akt signaling plays an important role in the events activated in response to angiogenic signals including basement membrane remodeling, tip cell migration and stalk cell proliferation [20–22]. Additionally, the activation of PI3K/Akt signaling can promote endothelial progenitor cell homing to ischemic tissue in the brain and heart, which is crucial for angiogenesis [23, 24].

Vascular endothelial growth factor (VEGF) is widely accepted as a primary inducer of angiogenesis [25]. Our previous results indicated that a VEGF/VEGFR mechanism may be involved in in vivo roxarsone-promoted angiogenesis [12]. Since PI3K/Akt activation mediates VEGF production and functions as a downstream signal of VEGF/VEGFR [26–28], the in vivo inter-relationship between VEGF and the PI3K/Akt pathway is complex, and it would be interesting to examine if and how these factors play a role in arsenic-induced angiogenesis.

Jiaqiao et al. [11] reported that roxarsone appeared to show the angiogenic potential in rat aorta endothelial cells by cell proliferation assays and tube formation assays. Only two events of angiogenesis were focused on in their research, which may not fully investigate the roles of roxarsone on different periods. In this study, the role of PI3K/Akt signaling in roxarsone-induced angiogenesis in rat vascular endothelial cells (ECs) was firstly investigated by evaluating growth, survival, migration and tube formation in these cells. Zhang et al. [12] reported the promotion effect of roxarsone on growth of the MCF-7 xenograft tumors. As MCF-7 cells were oestrogen-responsive and may not be optimal for investigating the effects of roxarsone on tumor growth so that a xenograft model of B16–F10 cells in C57BL/6 mice was investigated to understand the potential carcinogenic or tumor-promoting risk of roxarsone.

## Methods

### Experimental animals and chemicals

Male Wistar rats (weighing ~250 g) and C57BL/6 mice (weighing 20 ± 2 g) were obtained from the Center of Comparative Medicine, Yangzhou University, China. The animals were housed at 22 ± 2 °C and under 55 ± 5 % relative humidity with a regular 12-h light/dark schedule. Food and water were available ad libitum.

Roxarsone (Sigma-Aldrich, St. Louis, MO, USA) was dissolved in 5 mL methanol and then diluted to 50 mL with deionized water to obtain a 1.0 mM stock solution. The working solution of 0.01–10.0 µM roxarsone was made by further diluting the stock solution with incubation medium.

Sodium heparin and trypsin were purchased from Sigma-Aldrich. Dulbecco’s modified Eagle medium (DMEM), penicillin/streptomycin and fetal bovine serum (FBS) were purchased from Gibco (Invitrogen, Carlsbad, CA, USA). Recombinant rat VEGF_165_ was purchased from Peprotech CO. (Rocky Hill, NJ, USA). The PI3K inhibitor LY294002 was from Santa Cruz Biotechnology (Santa Cruz, CA, USA). Growth Factor Reduced Matrigel™ Matrix was purchased from BD Biosciences (San Jose, CA, USA). Rabbit monoclonal PI3K, phospho-PI3K, Akt and phospho-Akt antibodies were from Cell Signaling Technology (Danvers, MA, USA). Rabbit polyclonal VEGF antibody, 5-bromo-2-deoxyUridine (BrdU) and mouse monoclonal BrdU antibody were purchased from Boster Biotechnology (Wuhan, China).

### Isolation of rat thoracic aorta and primary EC culture

The Wistar rats were anaesthetized using 2 % thiopental sodium and sacrificed, and ECs were isolated from the thoracic aorta as described previously [11] and cultured in DMEM supplemented with 15 % (v/v) FBS, 100 µg/mL sodium heparin, 4 ng/mL VEGF and 100 U penicillin/streptomycin at 37 °C in a 5 % CO_2_ atmosphere. The cells were subcultured once they had formed a monolayer (after approximately 6 days of incubation). For the following assays, the ECs were digested with 2 % trypsin, briefly centrifuged at 1000 rpm for 10 min and resuspended at the required density in DMEM.

### Cell survival assessment by the MTT assay

Cells were plated in reduced serum and growth factor-containing DMEM (1:5 dilution of complete DMEM with non-supplemented DMEM) in 96-well plates at a cell density of 2 × 10^3^ cells/well, and incubated overnight. The wells were subsequently treated with PBS; 5 ng/mL VEGF; 0.1, 1.0 or 10.0 µM roxarsone; 20.0 µM LY 294002 or 20.0 µM LY 294002 plus 1.0 µM roxarsone for 24 h. After incubation, MTT reduction assays were performed at a final concentration of 0.4 mg/mL in medium. After 4 h, reduced formazan was solubilized with 150 µL dimethyl sulphoxide (DMSO) by shaking for 10 min, and absorbance was measured at 570 nm in a microplate reader (Multiskan MS, Labsystems, USA). The optical density (OD) rate was calculated by dividing the OD value of the test compounds by the OD values of the PBS control to evaluate the changes in cell viability caused by treatment. Six wells were replicated for each treatment.

### Cell proliferation assessment by the BrdU staining assay

Cells were seeded in 6-well plates at a cell density of 1 × 10^4^ cells/well in reduced serum and growth factor-containing DMEM and treated with the reagents as described above. After treatment for 24 h, BrdU was added at a final concentration of 30 µg/L in the medium, and incubated with the cells for 4 h. The cells were fixed with methanol containing 0.04 % H_2_O_2_ for 15 min; they were then incubated in 2 N hydrochloric acid at 37 °C for 1 h. Subsequently, the cells were blocked in 5 % bovine serum albumin (BSA) for 20 min at room temperature, and were then incubated overnight with the BrdU antibody (1:200 dilution) at 4 °C. After the first antibody was washed off, the cells were incubated with goat anti-mouse IgG-FITC (1:50 dilution) for 30 min at room temperature lucifugally. This was followed by excitation at 494 nm, after which BrdU-positive cells were counted under the Leica DMI 400B fluorescence microscope under 200× magnification.

### Cell migration assessment by the scratch assay

Cells were seeded in 6-well plates that were incubated in reduced serum and growth factor-containing DMEM till a monolayer was formed. The monolayer was then wounded by scratching with pipette tips and washed with PBS. The reagents described before were added to each well, and the wells were incubated at 37 °C in a 5 % CO_2_ atmosphere for 24 h. Cells were photographed at 100× magnification under a light microscope, and the distances to which they had migrated were quantified by subtracting the width of the scratch at 0 h from that at 24 h, measured by the Lecia QWin Pro V 3.5.1 software.

### Matrigel-induced tube formation assay

The Matrigel assay has been widely used for the in vitro measurement of tube formation of ECs and was performed as described previously [11]. Each well of 96-well plates was coated with 50 µL Matrigel, and the plates were incubated at 37 °C for 6 h to allow the Matrigel to polymerize fully. Cells were seeded at a density of 1.5 × 10^3^ per well into the prepared plates. Cells were treated as described before for 6 h. Images were acquired from five random fields of each well at a 200× magnification and were photographed using a Lecia inverted phase-contrast microscope. Endothelial tube formation was evaluated during the period of incubation. Capillary-like tube formation was quantified by counting the number of meshes in randomly selected images from each treatment group using Image-J with the Angiogenesis Analyze Plugin.

### Mouse B16–F10 xenograft studies

B16–F10 melanoma cells were provided by the Research Group of Laboratory Animals of Yangzhou University, and maintained in DMEM containing 10 % FBS and penicillin/streptomycin. Six-week-old C57BL/6 male mice weighing 20 g were housed at 22 ± 2 °C temperature, 55 ± 5 % relative humidity with 12 h light/dark schedule for 1 week of acclimatization. Then, 1 × 10^5^ B16–F10 cells in 0.3 mL nonsupplemented DMEM were injected subcutaneously into the external surface of the right ribs of the mice. The animals with visible tumors after cells injection 7 days, were intragastrically administered 0, 1, 5 or 25 mg/kg roxarsone in 100 µL PBS once a day for 1 week. During this week of administration, the body weight, feed and drink of the animals were assessed, and tumor measurement was conducted once a day. The length (L) and width (W) of tumors were measured using vernier calipers, and tumor volume was calculated using the formula L × W^2^. After the animals were sacrificed at 1 week administration, the tumors were excised, weighed, fixed overnight in neutral buffered formalin, embedded in paraffin, and sectioned. The sections were then stained with hematoxylin–eosin (H&E) and photographed using a light microscope at 100× magnification.

### Immunohistochemical staining for VEGF

Mouse tumor sections were de-paraffinized using traditional protocols and then incubated in 3 % peroxide for 10 min to inactivate endogenous peroxidase. Antigen retrieval was accomplished in 0.1 M sodium citrate brought to a boil in a microwave oven at high power, and then heating was continued at 30 % power for 10 min. Tissues were blocked with 5 % BSA, and incubated overnight at 4 °C with antibodies against VEGF (1:100 dilution). Then, the tissues were incubated with secondary antibody conjugated to horseradish peroxidase, and AEC peroxidase substrate (Boster, Wuhan, China). Sections were then counterstained with Mayor’s hematoxylin and photographed using a light microscope at 400× magnification.

### Western blot analysis of PI3K/Akt signaling and VEGF expression

Treated cells and tumor tissues were lysed in RIPA lysis buffer with phenylmethyl sulfonylfluoride (PMSF) and a phosphatase inhibitor cocktail (Beyotime, Shanghai, China) on ice. Then, the lysates were subjected to SDS-polyacrylamide gel electrophoresis with 20 µg protein loaded per lane. The gels were transferred to nitrocellulose (NC) membranes using a Bio-Rad Transblot apparatus. The membranes were blocked with 5 % BSA in TBS for 2 h at room temperature and then incubated overnight with the rabbit antibody against PI3K (1:1000 dilution), phospho-PI3K (1:1000 dilution), Akt (1:1000 dilution), phospho-Akt (1:1000 dilution), VEGF (1:200 dilution) and β-actin (1:250 dilution). Blots were developed using the enhanced chemiluminescence (ECL) detection system (Bio-Rad Laboratories). The intensity of the blots was measured using Image-J.

### Statistical analysis

The experiments were performed independently at least three times. The quantitative data are expressed as the mean ± standard deviation. Statistical analysis was performed using one-way analysis of variance (ANOVA) followed by the Bonferroni post-test unless otherwise noted, and p < 0.05 was considered to indicate statistical significance.

## Results

### Roxarsone stimulates EC survival, proliferation, migration and angiogenesis

**Fig. 1:**
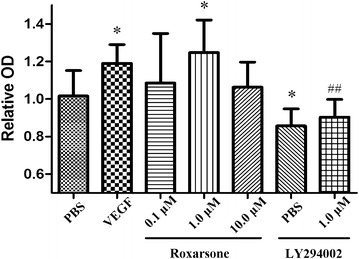
The effects of different treatments on the vitality of endothelial cells (ECs). The MTT assay was performed to evaluate the vitality of ECs treated with the indicated reagents for 24 h. The results presented are the mean ± SD values of three independent experiments relative to the PBS control. *p < 0.05 vs. the PBS control; ^##^p < 0.01 vs. 1.0 µM roxarsone

**Fig. 2:**
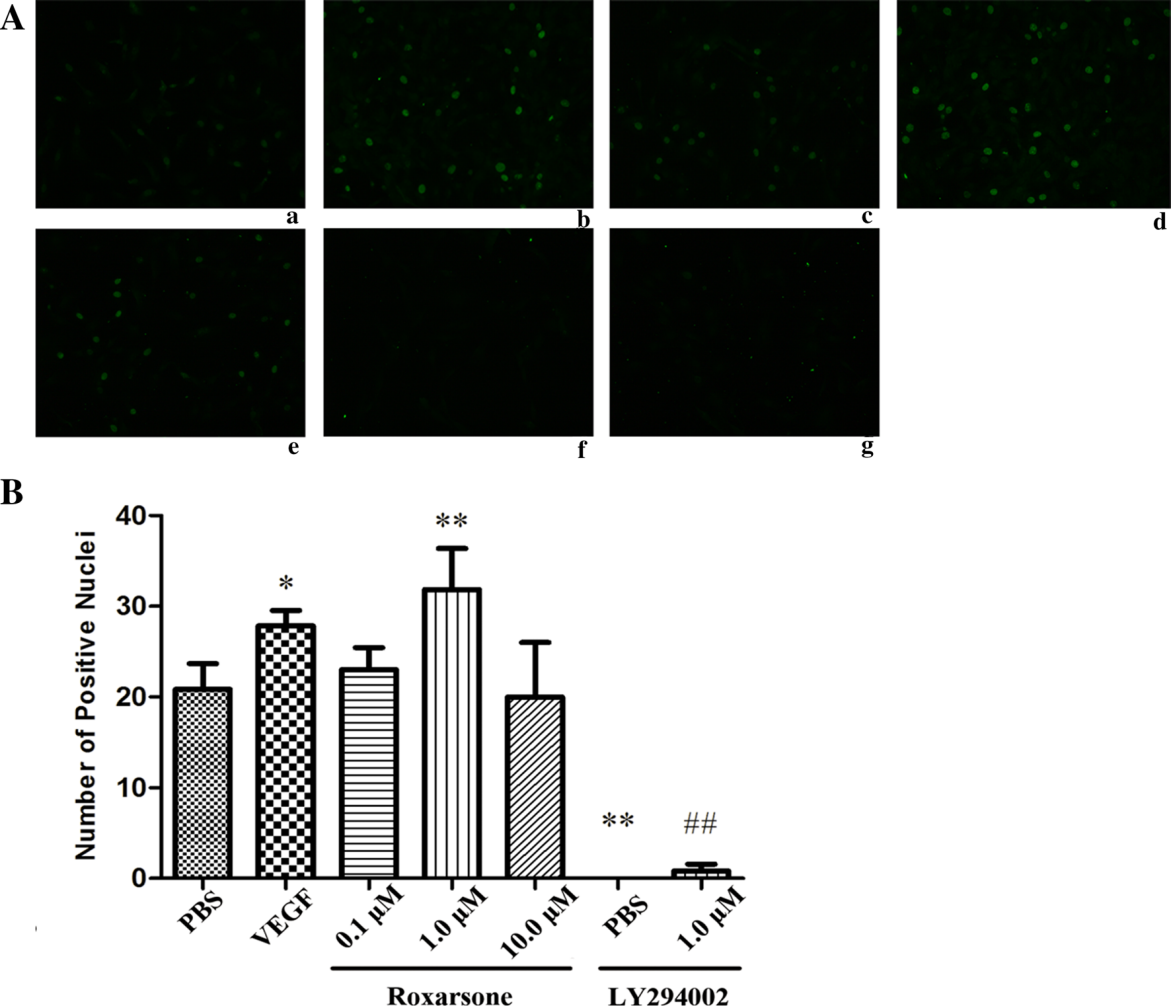
The effects of different treatments on the proliferation of endothelial cells (ECs). **A** The ECs treated with the indicated reagents for 24 h were subjected to BrdU staining (×200), and the number of positive ECs from at least five random fields per group were counted. Representative images are shown for the *a* PBS control; *b* VEGF; *c*–*e* 0.1, 1.0 and 10.0 µM roxarsone; *f* LY294002 and *g* LY294002 plus 1.0 µM roxarsone groups. **B** The number of positive cells are presented as the mean ± SD values from three independent experiments. *p < 0.05, **p < 0.01 vs. the PBS control; ^##^p < 0.01 vs. 1.0 µM roxarsone

**Fig. 3:**
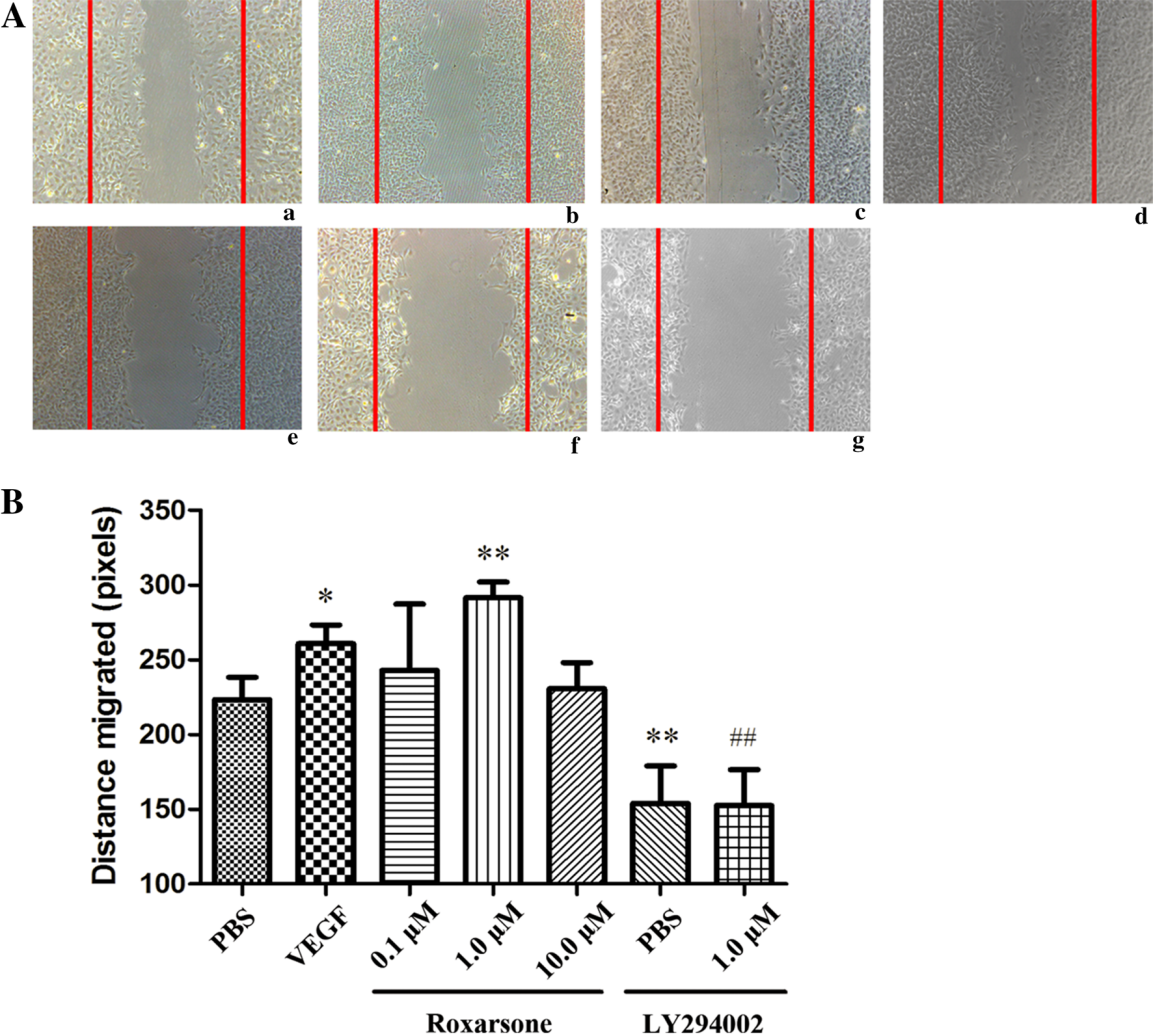
The effects of different treatments on the migration ability of endothelial cells (ECs). **A** ECs were wounded with a pipette and treated with the indicated reagents for 24 h. Representative images (×100) are shown for the *a* PBS control; *b* VEGF; *c*–*e* 0.1, 1.0 and 10.0 µM roxarsone, *f* LY294002 and *g* LY294002 plus 1.0 µM roxarsone groups. **B** The distance that the cells had migrated was calculated by subtracting the width of the scratch at 0 h from the width at 24 h. The results represent the mean ± SD values from three independent experiments. *p < 0.05, **p < 0.01 vs. the PBS control; ^##^p < 0.01 vs. 1.0 µM roxarsone

When ECs were incubated with 1.0 µM roxarsone and VEGF, the relative OD rate was significantly higher than that of the PBS control, as determined by the MTT assay (Fig. 1). The effect of roxarsone on EC proliferation was also determined by BrdU staining (Fig. 2). Treatment with 1.0 µM roxarsone and VEGF led to a significant increase in cell proliferation compared with the control cells. The scratch assay (Fig. 3) was performed to evaluate the impact of roxarsone on cell migration. The distance that the cells treated with 1.0 µM roxarsone had migrated was significantly longer than that travelled by the cells treated with the PBS control, and similar to that migration length of cells treated with VEGF.

**Fig. 4:**
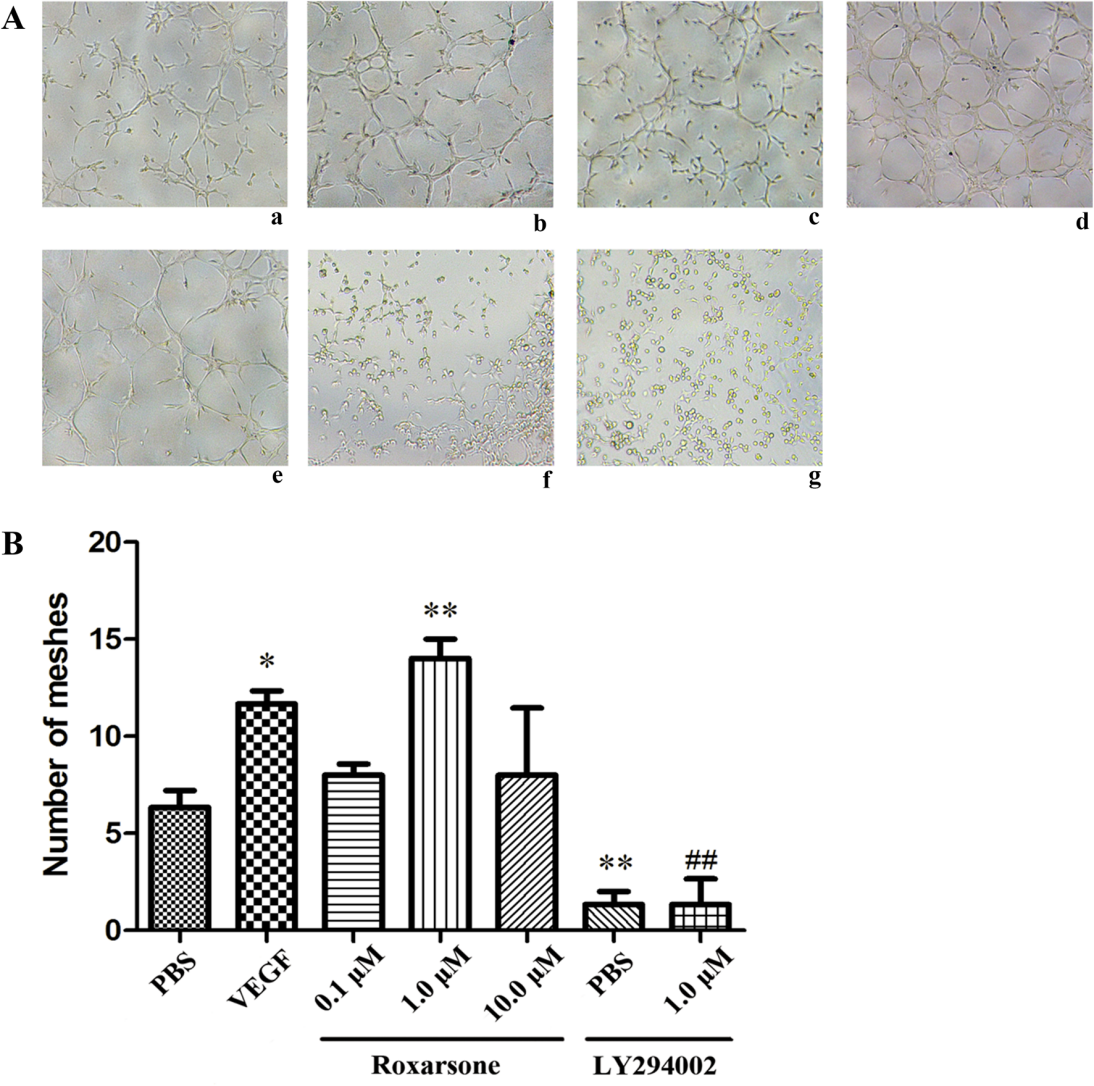
The effects of different treatments on endothelial cell (EC) tube formation. **A** After treatment with the indicated reagents for 6 h, tubular structure in each group was photographed at ×200 magnification. Representative images are shown for the *a* PBS control; *b* VEGF; *c*–*e* 0.1, 1.0 and 10.0 µM roxarsone, *f* LY294002 and *g* LY294002 plus 1.0 µM roxarsone groups. **B** The number of meshes in five randomly selected images for each group was measured using Image-J with the Angiogenesis Analysis Plugin. The results represent the mean ± SD values from three independent experiments. *p < 0.05, **p < 0.01 vs. the PBS control; ^##^p < 0.01 vs. 1.0 µM roxarsone

We next examined in vitro angiogenesis following stimulation of ECs with the reagents described before in Matrigel plates (Fig. 4). The results revealed that treatment with 1.0 µM roxarsone and VEGF significantly stimulated in vitro angiogenesis in ECs. However, treatment with 0.1 and 10.0 µM roxarsone did not have a statistically significant effect on any angiogenic parameters. Further, treatment with LY294002, a PI3K inhibitor, resulted in a significant reduction in vascular endothelial cell vitality, the number of BrdU-positive cells, the migration distance and the number of meshes in the tube formation assay. Treatment with LY294002 combined with 1.0 µM roxarsone led to a significant decrease in the angiogenic parameters compared to treatment with 1.0 µmol/L roxarsone alone.

### The PI3K/Akt/VEGF cascade is stimulated by roxarsone in ECs

**Fig. 5:**
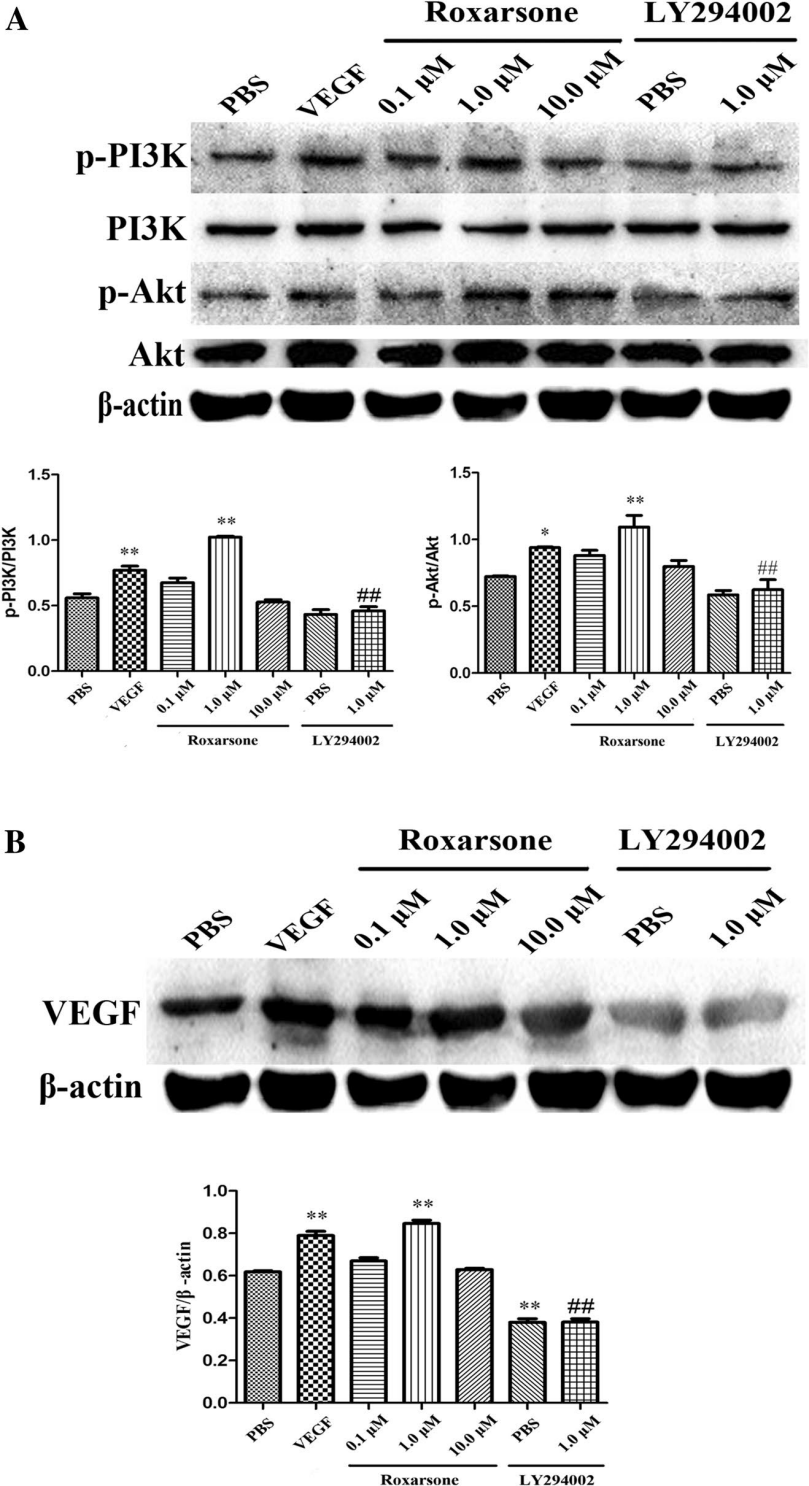
The effects of different treatments on activation of PI3K/Akt and expression of VEGF. Western blotting of total cell lysates treated with the indicated reagents for 1 h was performed, and β-actin was used as a loading PBS control. **A** The levels of PI3K and Akt phosphorylation were determined by phosphorylated proteins standardized to non-phosphorylated proteins. **B** The VEGF level of the cell lysates was determined by standardizing VEGF expression to β-actin expression. The results represent the mean ± SD values from three independent experiments. *p < 0.05, **p < 0.01 vs. the PBS control; ^##^p < 0.01 vs. 1.0 µM roxarsone

Since activation of PI3K/Akt is an important signaling event for angiogenesis, we examined whether roxarsone regulates phosphorylation of PI3K and Akt in ECs. We found that treatment with VEGF and 1.0 µM roxarsone significantly increased phosphorylation of PI3K and Akt (Fig. 5A) as well as VEGF expression (Fig. 5B). Further, LY294002 was found to suppress PI3K and Akt phosphorylation via its effect on VEGF expression. Phosphorylation of PI3K and Akt and the expression of VEGF in ECs treated with LY294002 plus 1.0 µM roxarsone were significantly lower than that in ECs treated with 1.0 µM roxarsone. These results suggest that the PI3K/Akt signaling pathway may be stimulated by roxarsone and required for roxarsone-induced VEGF expression in ECs.

### Roxarsone promotes the growth of mouse B16–F10 xenografts

**Fig. 6:**
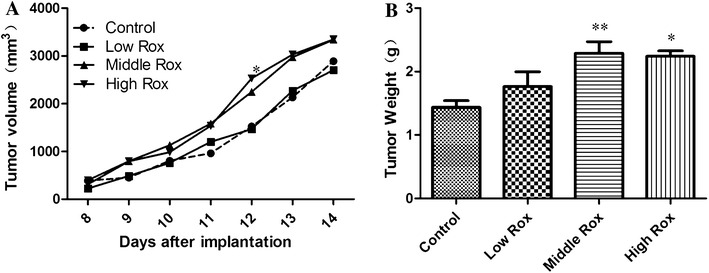
Roxarsone promotes the growth of B16–F10 xenografts growth. B16–F10 tumor cells (1 × 10^5^) were implanted subcutaneously into the external surface of the right ribs of mice. After 7 days, the mice were intragastrically administered 0, 1, 5 or 25 mg/kg roxarsone once a day for another 7 days; the corresponding groups are designed as the Control, Low Rox, Middle Rox and High Rox groups respectively. **A** Tumor size was measured using vernier calipers, and tumor volume was calculated as described in “Methods” section. Data represent the average tumor volumes at the given day after implantation. Significant growth was observed in all the groups (p < 0.05, n = 6), and significant differences were observed between treatment groups and the PBS control group (*p < 0.05, as determined by two-way ANOVA). **B** B16–F10 xenografts were excised and weighed. The results represent the mean ± SD values of six mice. *p < 0.05, **p < 0.01 vs. the PBS control

**Fig. 7:**
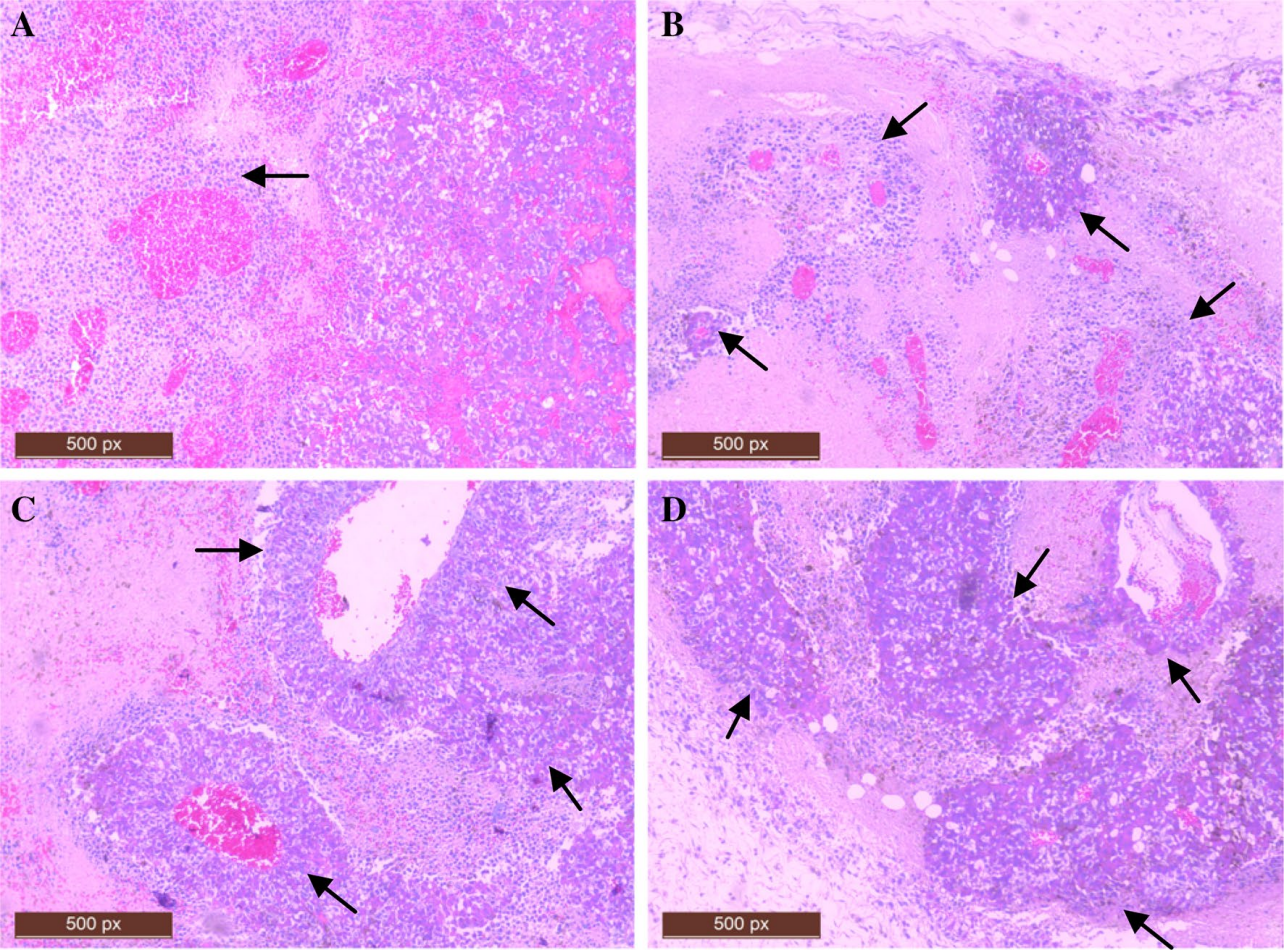
Roxarsone affects the growth pattern of B16–F10 xenografts. The xenograft tumors were excised; paraffin sections were created and stained with H&E staining and histologically analyzed (×100). Representative images (of at least five mice per group) are shown for the **A** PBS control and **B**–**D** 1, 5, and 25 mg/kg roxarsone groups designed as Control, Low Rox, Middle Rox and High Rox. Clustered growth with centrally located blood vessels (indicated by the arrow) was observed in the roxarsone groups, but this was rarely observed in the PBS control group

From the day of intragastric administration of 0, 1, 5 or 25 mg/kg roxarsone, the body weights of the mice changed, decreasing first and then increasing, but there was no significant difference between treatment groups (data not shown). Additionally, none of the mice showed any abnormal nervous symptoms. Surface measurement indicated that tumor growth occurred in all of the mice in the study, regardless of which treatment group they belonged to (Fig. 6A). However, mice that received 25 mg/kg of roxarsone had significantly heavier (Fig. 6B) and larger tumors than the untreated mice. Mice treated with 5 mg/kg roxarsone also had heavier and larger tumors, but the difference was not significant compared to the untreated group. Staining of paraffin sections of 2-week-old tumors stained with H&E (Fig. 7) indicated that there were changes in the growth pattern of tumor cells and blood vessel size, according to the roxarsone dose administered. As seen in Fig. 7, the B16–F10 cells in the xenograft tumors of untreated mice were typically diffuse, without a distinct growth pattern. The structure and organization of the xenograft tumors in the 5 and 25 mg/kg roxarsone groups appeared tight, and the B16–F10 cells showed inward cluster growth that was centered on a blood vessel. In contrast, the structure of the xenograft tumors of mice treated with 1 mg/kg roxarsone was similar to that of untreated mice. Additionally, the xenografts in mice administered 5 and 25 mg/kg roxarsone contained a high number of vessels and vessels of a larger diameter.

### Roxarsone induces activation of PI3K/Akt signaling and VEGF expression

**Fig. 8:**
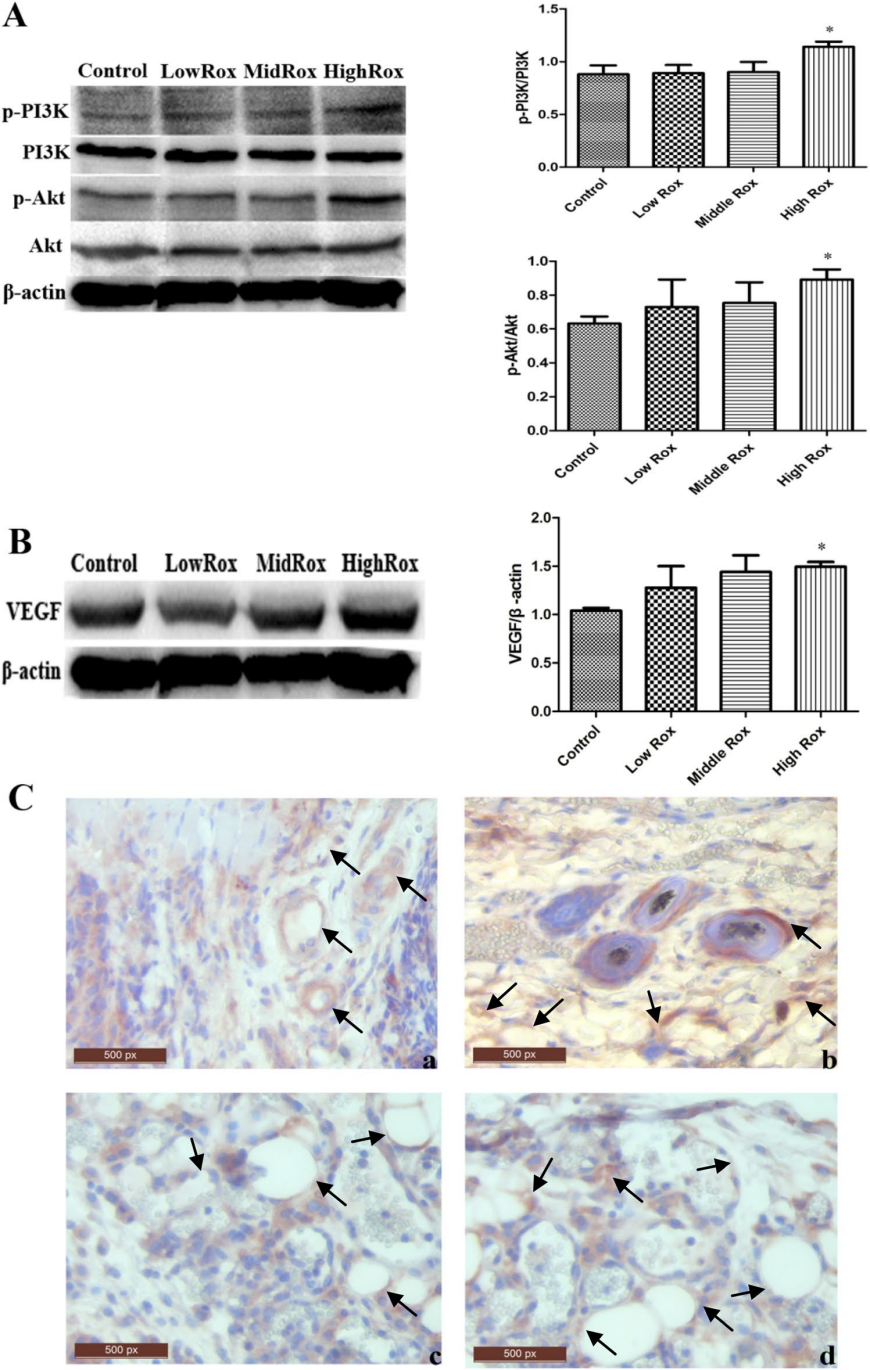
Roxarsone promotes activation of PI3K/Akt and expression of VEGF in B16–F10 xenografts. Western blotting of total tumor lysates was performed with β-actin used as a loading PBS control. The groups designed as Control, Low Rox, Middle Rox and High Rox represent the mice that were administered 0, 1, 5 and 25 mg/kg roxarsone respectively. **A** The levels of PI3K and Akt phosphorylation were determined by phosphorylated proteins standardized to non-phosphorylated proteins. **B** The VEGF level of tumors was determined by VEGF expression standardized to β-actin expression. The results represent the mean ± SD values from three independent experiments. *p < 0.05 vs. the PBS control. **C** The xenograft tumors were excised, and paraffin sections were subjected to VEGF immunochemical analysis (×400). Representative images (of at least five mice per group) are shown for the *a* PBS control and *b*–*d* 1, 5 and 25 mg/kg roxarsone groups. Blood vessels surrounding the positive staining regions (arrow indicated) were observed in the PBS control and roxarsone groups

Recent evidence has indicated that the dysregulation of PI3K signaling promotes tumorigenesis and angiogenesis in various cancer types [18, 19, 29]. Therefore, in the present study, phosphorylation of PI3K/Akt signaling in the B16–F10 xenografts of mice that were administered roxarsone was examined using western blot analysis. We found that treatment with 25 mg/kg roxarsone resulted in a significant increase in phosphorylation of PI3K and Akt when compared with untreated mice (Fig. 8A). Similarly, expression of VEGF, an index related to clinical outcome in cancer patients [24], in the B16–F10 xenografts of mice that were administered 25 mg/kg roxarsone was significantly higher than that of untreated mice; this was also observed in our western blot results (Fig. 8B). Immunohistochemical analysis of VEGF demonstrated that the xenografts in untreated and 1 mg/kg roxarsone-treated mice contained a low number of vessels surrounded by positive staining, whereas the xenografts in the 5 and 25 mg/kg roxarsone groups contained a greater number of vessels (Fig. 8C).

## Discussion

In this study, we used rat endothelial cells and a B16–F10 xenograft model to determine (1) whether exposure to roxarsone induces angiogenesis, (2) whether PI3K/Akt signaling is involved in roxarsone-induced angiogenesis, and (3) whether roxarsone promotes tumor growth.

Vessel sprouting (angiogenesis) is an extensively studied aspect of vascularization. Upon activation of ECs by proangiogenic molecules, cell–cell junctions and the basement membrane are remodeled in tandem with pericyte detachment, which allows tip cells to migrate in response to signals. The sprout then elongates through the proliferation of stalk cells, which form a lumen and recruit pericytes for stabilization [29]. In a previous MTT assay or tube formation assay, roxarsone showed a non dose-depended effect on angiogenesis [11, 12], which appeared to have peak value of stimulating effects at 1 µM levels and slightly decreased in the stimulating effects at high levels (1–10 µM). The data presented here suggest that roxarsone not only increased EC vitality (Fig. 1) and promoted tube formation in Matrigel (Fig. 4), but also enhanced EC proliferation (Fig. 2) and migration (Fig. 3), based on the findings of the BrdU assay and scratch assay respectively. These are rarely reported but not trivial events associated with angiogenesis. Additionally, the effects of roxarsone were concentration dependent and observed in the concentration range of 0.01–1.0 µM. Although no obvious inhibitory effect was observed when 10.0 µM of roxarsone was used, the pro-angiogenic effect was indeed weakened at this concentration. It was without a doubt that the dosage range we used here was narrow while interval was broad, which limited the precise investigation in dose-effect relationship of roxarsone.

PI3K/Akt signaling is involved in multiple signaling pathways in the regulation of cell proliferation, differentiation, survival and migration [30], and it is closely associated with the occurrence and development of angiogenesis. To determine whether PI3K/Akt signaling was required for the effects of roxarsone on ECs, we assessed PI3K/Akt signaling by western blot analysis. The results indicated that roxarsone indeed triggered phosphorylation of PI3K and Akt (Fig. 5A). Further, incubation of ECs with the PI3K inhibitor LY294002 abrogated activation of ECs induced by roxarsone via decrease in the phosphorylation of PI3K and Akt. These findings indicate that activation of PI3K/Akt signaling is required for roxarsone-induced angiogenesis. In the present study, we also found that roxarsone induced an increase in the expression of VEGF (Fig. 5B), which is a key factor in the induction of angiogenesis. Inhibition of PI3K/Akt signaling abrogated the increase in VEGF expression induced by roxarsone, which suggests that roxarsone triggered the PI3K/Akt/VEGF signaling cascade. Our current results are consistent with previous reports [31, 32]. However, we did not examine how PI3K/Akt signaling triggered by roxarsone regulates VEGF expression. Wang et al. [33] showed that arsenic increased HIF-1α expression through PI3K/Akt signaling to induce VEGF expression in an oxygen-independent manner in human uroepithelial cells. Additionally, Tsai et al. [34] demonstrated that inhibitor of DNA binding 1 (Id1) was involved in the arsenic-promoted angiogenesis via PI3K/Akt/NF-κB signaling. Arsenite treatment decreased the activity of GSK3 [35], and also played the role of a downstream effector of PI3K/Akt, which caused β-catenin accumulation in the nucleus and VEGF expression [36]. Interestingly, Wang et al. [37] reported that arsenic exposure activated PI3K/Akt signaling to induce GSK3 phosphorylation in DLD-1 and HLT116 cells, while Piyajit et al. [35] demonstrated that arsenite inhibited the activity of GSK3 without activating its upstream kinase Akt. Thus, some of the findings reported in the literature so far are controversial. Given that the mechanism for arsenic-induced promotion of VEGF expression through PI3K/Akt signaling is complicated, the mechanisms underlying the effects of roxarsone remain to be investigated in the future.

For many years, arsenic has been related to various types of cancer, but there is very little evidence for the direct carcinogenic effect of arsenic [38, 39]. Instead, the tumor-promoting effect of arsenic appears to be enhanced in the presence of other carcinogens such as UV radiation [40]. In fact, the present and previous research on the B16–F10 xenograft model and the MCF-7 xenograft model [12] has only provided evidence for the tumor-enhancing effect of roxarsone when it is present as a co-carcinogen. Nicole et al. [8] demonstrated that significant tumor growth was observed in all animals that were given biweekly injections of 0.5–5.0 mg/kg As(III), with the largest tumors occurring in animals treated with lower doses of As(III). The present results indicate that 25 mg/kg roxarsone, a far higher dose than that used in the Nicole et al. study, significantly enhanced B16–F10 xenograft growth (Fig. 6). The similarity in the effects of these arsenic compounds even though they were administered at different dosages may be related to differences in the mode of administration: that is, intraperitoneal administration may be associated with higher plasma concentration and longer maintenance time than oral administration [41].

In the present study, the H&E staining (Fig. 7) and VEGF immunohistochemical staining (Fig. 8C) results indicated that roxarsone was capable of inducing tumor angiogenesis, which is an important process in the development of most tumors and might further explain the co-carcinogenicity of roxarsone. IHC analysis of VEGF in tumors might be a feasible method for assessing angiogenesis when the blood vessels of tumors are immature with the intact lumen of ECs invisible in H&E staining.

Vasculogenic mimicry (VM), which was observed in melanomas by Maniotis [42], has been widely recognized as a novel indicator of tumor microcirculation [43, 44]. The mechanism of VM may be attributed to tumor cell transformation and matrix remodeling [45]. Notably, VEGF has an impact on the formation of VM, and cells with high expression of VEGF appear to have the potential to form VM [46, 47]. The relative expression of VEGF in xenografts as measured via western blot analysis (Fig. 8B) also indicated that high levels of VEGF expression were closely associated with the growth of xenografts, in which microcirculation was more obvious (Figs. 7, 8C) in mice exposed to high doses of roxarsone.

Recent studies have highlighted the importance of the PI3K/Akt axis in various types of tumor cells. PI3K/Akt signaling is implicated in different processes that are characteristic of cancer including growth signal autonomy, insensitivity to antiproliferation signals, inhibition of apoptosis, angiogenesis, invasion and metastasis [48]. In the current study, western blot analysis of B16–F10 xenografts showed that the phosphorylation level of PI3K/Akt increased with the dose of roxarsone administered (Fig. 8A). Although these data are still preliminary and need to be confirmed in more detailed experiments, they nonetheless demonstrate that a PI3K/Akt mechanism may be involved in the development of B16–F10 xenografts induced by roxarsone.

## Conclusions

Altogether, our findings suggest that roxarsone promoted EC vitality, proliferation, migration and tube formation in vitro and B16–F10 xenograft growth with pro-angiogenic potential in vivo. Importantly, PI3K/Akt signaling played a role in the roxarsone-induced angiogenesis. Thus, understanding the angiogenic impact of roxarsone and its mechanism would shed light on the potential safety and risks of using arsenic compounds in human beings.

## Abbreviations

Rox: roxarsone
PI3K: phosphatidylinositol3-kinase
PIP2: phosphatidylinositol-(4,5)-bisphosphate
PIP3: phosphatidylinositol-(3,4,5)-triphosphate
Akt: protein kinase B
mTOR: mammalian target of rapamycin
BAD: BclxL/Bcl-2-associated death promoter
Bcl-2: B-cell lymphoma/leukemia-2
MDM2: murine double minute
NF-κB: nuclear factor κB
VEGF: vascular endothelial growth factor
eNOS: endothelial NO synthase
HIF: hypoxia inducible factor
LY294002: PI3K inhibitor
ROS: reactive oxygen system
ECs: vascular endothelial cells
MVD: micro-vessel density
DMSO: dimethyl sulphoxide
DMEM: Dulbecco’s modified eagle medium
FBS: foetal bovine serum
MTT: 3-(4,5-dimethyl-2-thiazolyl)-2,5-diphenyl-2-*H*-tetrazolium bromide
PBS: phosphate buffered saline
BSA: albumin from bovine serum
TBS/TBST: tris-buffered saline (with Tween 20)
OD: optical density
H&E: hematoxylin–eosin
PMSF: phenylmethyl sulfonylfluoride
NC: nitrocellulose
ANOVA: analysis of variance
SD: standard deviation
